# Dopamine Induced Neurodegeneration in a PINK1 Model of Parkinson's Disease

**DOI:** 10.1371/journal.pone.0037564

**Published:** 2012-05-25

**Authors:** Sonia Gandhi, Annika Vaarmann, Zhi Yao, Michael R. Duchen, Nicholas W. Wood, Andrey Y. Abramov

**Affiliations:** 1 Department of Molecular Neuroscience, UCL Institute of Neurology, London, United Kingdom; 2 Department of Cell and Developmental Biology, UCL, London, United Kingdom; Virginia Commonwealth University, United States of America

## Abstract

**Background:**

Parkinson's disease is a common neurodegenerative disease characterised by progressive loss of dopaminergic neurons, leading to dopamine depletion in the striatum. Mutations in the PINK1 gene cause an autosomal recessive form of Parkinson's disease. Loss of PINK1 function causes mitochondrial dysfunction, increased reactive oxygen species production and calcium dysregulation, which increases susceptibility to neuronal death in Parkinson's disease. The basis of neuronal vulnerability to dopamine in Parkinson's disease is not well understood.

**Methodology:**

We investigated the mechanism of dopamine induced cell death in transgenic PINK1 knockout mouse neurons. We show that dopamine results in mitochondrial depolarisation caused by mitochondrial permeability transition pore (mPTP) opening. Dopamine-induced mPTP opening is dependent on a complex of reactive oxygen species production and calcium signalling. Dopamine-induced mPTP opening, and dopamine-induced cell death, could be prevented by inhibition of reactive oxygen species production, by provision of respiratory chain substrates, and by alteration in calcium signalling.

**Conclusions:**

These data demonstrate the mechanism of dopamine toxicity in PINK1 deficient neurons, and suggest potential therapeutic strategies for neuroprotection in Parkinson's disease.

## Introduction

Mitochondrial dysfunction plays a major role in the pathogenesis of Parkinson's disease (PD), and has been demonstrated in mendelian PD models, toxin based PD models, and studies of sporadic PD brain tissue [1], [2]. One of the key models in characterising mitochondrial pathology in PD has been based on loss of PINK1 function. Mutations in the PINK1 gene cause an autosomal recessive form of PD [3]. PINK1 is a mitochondrial kinase that exerts a neuroprotective function. Although the substrates of PINK1 are not established, Drosophila and mammalian models of PINK1 deficiency have demonstrated significant mitochondrial abnormalities in the form of aberrant fission-fusion, loss of cristae, and mitochondrial swelling [4], [5]. We have previously studied mitochondrial physiology associated with PINK1 deficiency and demonstrated impaired calcium homeostasis, resulting in mitochondrial calcium overload and reduced threshold for calcium-induced opening of the permeability transition pore (PTP). In addition, we have shown that respiration is impaired in PINK1 deficient cells due to the reduced availability of substrates for the respiratory chain. As a result of the impaired bioenergetic function and calcium homeostasis, PINK1 deficient mitochondria have lower mitochondrial membrane potential, and higher levels of mitochondrial and cytosolic ROS production. Together this mitochondrial dysfunction may account for the reduced viability of PINK1 deficient neurons with aging [6], and increased susceptibility to apoptosis.

Although this mitochondrial pathophysiology exists in all neurons in the brain, neuronal death in Parkinson's disease is specific for certain brain regions. In the early stages of sporadic Parkinson's disease, one of the pathological hallmarks is the loss of substantia nigra pars compacta (SNpc) dopaminergic neurons, although as the disease progresses, non-dopaminergic neurons eventually become affected. Indeed, the initial selectivity of dopaminergic neurons remains a fundamental question in PD biology. Dopaminergic neurons are neurons that synthesise, package and release dopamine, and are thus exposed to intracellular and extracellular dopamine. Therefore it has been suggested that dopamine itself may be the cause of the selective cellular vulnerability in PD. However the interaction between mitochondrial dysfunction and sensitivity to dopamine has not yet been shown in genetic models of PD, and therefore it is unclear how mitochondrial dysfunction may particularly render dopaminergic neurons vulnerable to cell death.

In this study we have investigated the effect of dopamine in a model of mitochondrial dysfunction in PD induced by PINK1 deficiency. We have previously reported that dopamine induces a cytosolic calcium signal in astrocytes and neurons through receptor- independent mechanisms [7], [8]. Here we demonstrate that the dopamine induced calcium signal has detrimental consequences in cells with impaired mitochondrial function. Dopamine increases mitochondrial calcium concentration, increases ROS production and precipitates mPTP opening, leading to cell death in vulnerable neurons. This work explains why neurons with mitochondrial dysfunction that are exposed to dopamine may be particularly susceptible to cell death in PD. Furthermore based on the mechanism of dopamine induced cell death, we have proposed novel strategies for neuroprotection.

## Results

### Dopamine induces mitochondrial depolarisation in PINK1 KO cells

**Figure 1:**
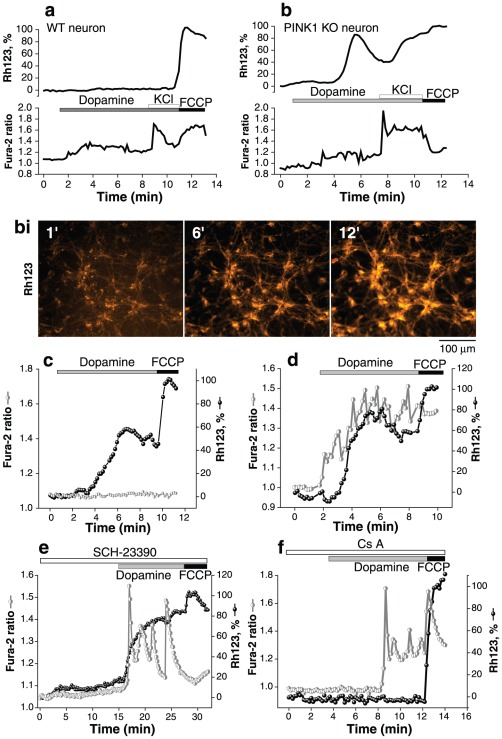
The effect of dopamine on Δψ_m_ and [Ca^2+^]_c_ in midbrain neurons and astrocytes. a: Dopamine 20 µM induces a modest rise in [Ca^2+^]_c_ in wt neurons and astrocytes, as measured by fura-2 ratio. There are no associated changes in Δψ_m_, as measured by Rhodamine 123 fluorescence. Depolarisation of the plasma membrane using 50 mM KCl induces opening of voltage sensitive calcium channels and a further rise in [Ca^2+^]_c_, with no change in ΔΨ_m_. b: Dopamine 20 µM induces a larger rise in [Ca^2+^]_c_ in PINK1 ko cells. Furthermore this increase in [Ca^2+^]_c_ is associated with a reduction in ΔΨ_m_. Application of 50 mM KCl induces a further rise in [Ca^2+^]_c_ and profound mitochondrial depolarisation. Figure bi demonstrates the increase in Rh123 fluorescence at 1 minute, 6 minutes and 12 minutes that reflects mitochondrial membrane depolarisation in PINK1 ko cells. c, d: Single cell traces of PINK1 ko neurons demonstrating that dopamine induced decrease in ΔΨ_m_ occurs independently of any change in [Ca^2+^]_c_ in some cells (Figure 1c), whereas in other cells dopamine induced decrease in ΔΨ_m_ co-incides with a rise in [Ca^2+^]_c_ (Figure 1d). e: Incubation with D1/D5 receptor antagonist SCH23390 (20 µM) did not alter the dopamine induced calcium signal or mitochondrial depolarisation in PINK1 ko neurons. f: Dopamine induced mitochondrial depolarisation was prevented by application of the PTP inhibitor 1 µM cyclosporine A.

We investigated the effect of dopamine on [Ca^2+^]_c_ and mitochondrial membrane potential (Δψ_m_) in postnatal midbrain co-cultures of astrocytes and neurons from wildtype (wt) and PINK1 knockout (ko) mice. In control cells, application of 20 µM dopamine induced small but significant sporadic changes in [Ca^2+^]_c_ (Fura-2 ratio rose from 1.11±0.02 to 1.34±0.05; n = 154 for neurons; p<0.05; from 1.01±0.02 to 2.1±0.1 for astrocytes; n = 197). This was not associated with any change in Δψ_m_ (Fig. 1a). Application of 50 mM KCl resulted in a further increase in [Ca^2+^]_c_ caused by depolarisation of the plasma membrane and opening of voltage gated calcium channels in neurons. Again no change in Δψ_m_ was detected in control neurons (Fig. 1a), confirming normal mitochondrial function in these cells [6], [9], [10]. In contrast to wt cultures, application of dopamine to the majority of PINK1 ko neurons (n = 101 of 167) and a subset of astrocytes (n = 79 of 214) was associated with profound mitochondrial depolarisation (rhodamine 123 (Rh123) signal rose to >80%, Figure 1b; average rise in Rh123 signal is 58.8±14%; p<0.001). The mitochondrial depolarisation was associated with a rise in [Ca^2+^]_c_ in 64% neurons (Figure 1d), but also occurred in the absence of any detectable change in [Ca^2+^]_c_ in 46% neurons (Figure 1c). We have previously reported that dopamine is able to induce a calcium signal in both astrocytes and neurons using a receptor independent mechanism [7], [8]. The dopamine induced calcium signal in PINK1 ko neurons and astrocytes was confirmed also to be receptor independent as it was not blocked either in the presence of the D1/D5-like antagonist (20 µM SCH-23390) (n = 39; Fig. 1e) or by the D2-like receptor antagonist (20 µM sulpiride) (n = 45). Pre-incubation of cells with dopamine receptor antagonists also had no effect on the dopamine induced mitochondrial depolarisation in PINK1 ko cells (Fig. 1e).

The dopamine induced decrease in Δψ_m_ (but not the dopamine induced calcium signal) was prevented by pre-incubation with a PTP inhibitor, Cyclosporin A (0.5 µM) (dopamine-induced depolarisation reduced from 58.8±14% to 18.9±1.3%; Figure 1c, n = 64 neurons; n = 122 astrocytes, Figure 1f). Thus non-toxic concentration of dopamine is able to induce PTP opening in PINK1 ko cells, but not in control cells. This led us to investigate the two major factors that influence the induction of PTP opening: (1) Mitochondrial calcium overload and (2) overproduction of reactive oxygen species.

### Dopamine induces changes in mitochondrial calcium in PINK1 KO cells

**Figure 2:**
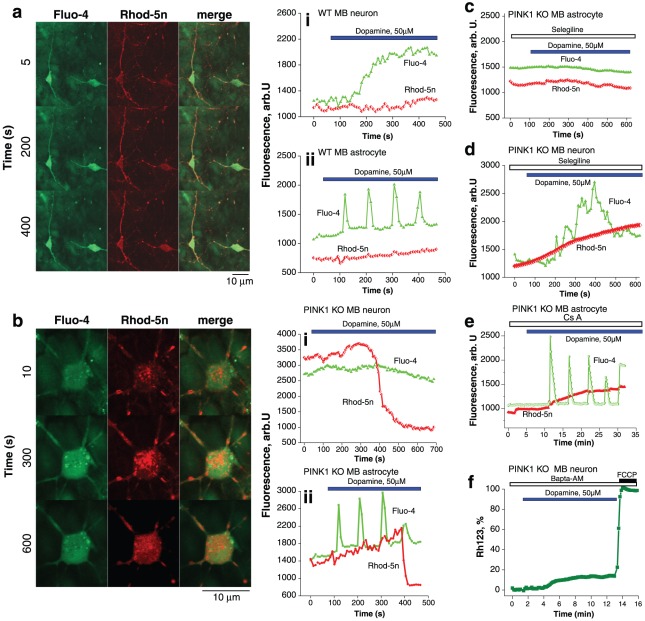
The effect of dopamine on [Ca^2+^]_c_ and [Ca^2+^]_m_. a: Image demonstrates two wt midbrain neurons on a bed of astrocytes loaded with fluo-4 (green) and rhod-5n (red). Application of dopamine induced an increase in the fluo-4 fluorescence with minor change in the rhod-5n fluorescence. (ai) A single neuron demonstrating the dopamine induced rise in [Ca^2+^]_c_ (measured by fluo-4, green) with no concomitant rise in [Ca^2+^]_m_ (measured by rhod-5n, red). (aii) A single astrocyte demonstrating the dopamine induced oscillations in [Ca^2+^]_c_ with no concomitant rise in [Ca^2+^]_m_. b: Image demonstrates a PINK1 ko midbrain neuron loaded with fluo-4 and rhod-5n. Dopamine induced initially a significant increase in the rhod-5n fluorescence, indicating mitochondrial calcium overload. This is followed by rapid redistribution of the rhod-5n dye from the mitochondria to the cytosol, representing mitochondrial permeability transition pore opening. (bi) Trace of single PINK1 ko midbrain neuron demonstrating an initial high level of rhod-5n fluorescence suggesting higher [Ca^2+^]_m_. There is an increase in the [Ca^2+^]_m_ followed by rapid reduction reflecting PTP opening. No change in [Ca^2+^]_c_ is detected. (bii) Trace of single PINK1 ko midbrain astrocyte demonstrating dopamine induced oscillations in [Ca^2+^]_c_, associated with an increase in [Ca^2+^]_m_, followed by PTP opening. c,d: The effect of selegiline on dopamine induced calcium signal: in PINK1 ko astrocytes (c), application of selegiline abolished the dopamine induced rise in [Ca^2+^]_c_ and [Ca^2+^]_m_. In PINK1 ko neurons (d), application of selegiline did not abolish the dopamine induced rise in [Ca^2+^]_m_. e: Application of CsA did not affect the dopamine induced rise in mitochondrial calcium, but did prevent the rapid decline in rhod-5n signal induced by PTP opening. f: Dopamine induced mitochondrial depolarisation in PINK1 ko neurons was blocked by addition of a calcium chelator BAPTA-AM 50 Mm.

Cells were loaded with Rhod-5n and fluo-4 to simultaneously measure [Ca^2+^]_m_ and [Ca^2+^]_c_ respectively. Application of dopamine 50 µM to wt neurons and astrocytes induced a small and sustained increase in [Ca^2+^]_c_ (fluo-4 fluorescence signal rose by 640±56 arb. U. for neurons (n = 56 neurons; Figure 2ai, ii); fluo-4 signal rose by 750±80 arb.U; n = 127 astrocytes). In wt neurons and astrocytes there were non significant changes in the Rhod-5n fluorescence ([Ca^2+^]_m_).

We have previously performed an indirect quantification of the basal level of mitochondrial calcium and demonstrated that PINK1 ko is associated with higher basal levels of mitochondrial calcium [6]. Using the non-ratiometric dye rhod-5n we observed again that the basal level of [Ca^2+^]_m_ appeared higher in PINK1 ko neurons than in wt neurons (n = 87; Figure 2b). Interestingly in PINK1 ko neurons exposed to dopamine, there was an initial rise in mitochondrial rhod-5n fluorescence (Figure 2bi). The rise in mitochondrial calcium was followed by the rapid disappearance of the rhod-5n signal within single mitochondria associated with PTP opening and the redistribution of the dye to the cytosol. This was not observed in wt cells in Figure 2a.

In PINK1 ko astrocytes, application of dopamine induced [Ca^2+^]_c_ oscillations (fluo-4 signal rose by 1435±124 arb. U.) which were associated with an increase in [Ca^2+^]_m_, which eventually led to a rapid decrease of mitochondrial rhod-5n signal (n = 112; Figure 2bii.). This is due to the opening of the PTP, as it was prevented by incubation with CsA. Figure 2e In another study we reported the mechanism of the dopamine-induced calcium signal in astrocytes, and demonstrated that it could be blocked by application of an inhibitor of monoamine oxidase (MAO) – 20 µM selegiline [8]. In PINK1 ko astrocytes 20 µM selegiline completely blocked both [Ca^2+^]_c_ and [Ca^2+^]_m_ changes (n = 67; Figure 2c). The inhibitor of MAO did not prevent against the dopamine induced rise in cytosolic or mitochondrial calcium in PINK1 ko neurons (n = 54; Figure 2d). However selegiline did prevent the PTP opening associated with dopamine in PINK1 ko neurons, and thus prevent the loss of the rhod-5n signal seen in Fig. 2b.

In order to test whether the dopamine induced rise in mitochondrial calcium was responsible for dopamine induced PTP opening, cells were incubated with the calcium chelator Bapta-AM which rapidly penetrates both the cytosol and mitochondria and buffers [Ca^2+^]_c_ and [Ca^2+^]_m_. 10 min preincubation of PINK1 ko midbrain neurons with 50 µM Bapta-AM prevented the dopamine induced Δψ_m_ depolarisation (the dopamine-induced depolarisation was reduced from 58.8±14% to 17.8±1.4%; n = 52; p<0.001; Figure 2f).

### Dopamine induces a rise in ROS in PINK1 KO and WT neurons

**Figure 3:**
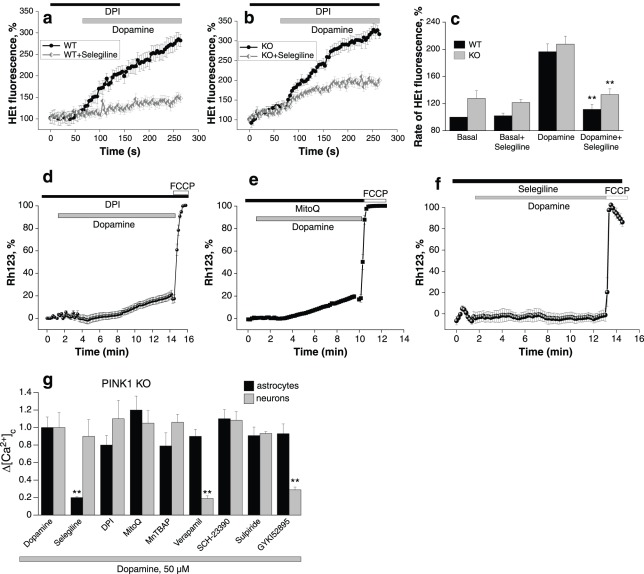
The role of ROS in dopamine induced mitochondrial depolarisation in PINK1 ko neurons. a: Dopamine induces a rise in cytosolic ROS production in wt neurons, in the presence of inhibitor of NADPH oxidase, 0.5 µM DPI. Dopamine induced ROS production was prevented by application of the MAO inhibitor, 20 µM selegiline. b: Dopamine induced a similar rise in ROS production in PINK1 ko neurons, which is partially inhibited by the application of MAO inhibitor, selegiline. c: Histogram demonstrating the increase in ROS production in PINK1 ko neurons compared to wt neurons, and the dopamine induced increase in ROS in both wt and PINK1 ko neurons. The dopamine induced increase in ROS may be reduced in both wt and PINK1 ko neurons by application of selegiline. d: Application of an inhibitor of NADPH oxidase, DPI, partially prevented dopamine induced mitochondrial depolarisation in PINK1 ko neurons. e: Application of mitochondrially located antioxidant, MitoQ, partially prevented dopamine induced mitochondrial depolarisation in PINK1 ko neurons. f: Application of MAO inhibitor, selegiline, completely prevented the dopamine induced mitochondrial depolarisation in PINK1 ko neurons. g: Histogram demonstrating the effect of antioxidants, verapamil, and dopamine receptor antagonists on the dopamine-induced calcium signal in PINK1 ko astrocytes and neurons. The effect of dopamine on the calcium signal (fura-2 ratio) was normalised to 1.0. Selegiline has a selective effect in reducing the calcium signal in astrocytes, while verapamil has a selective effect in reducing the calcium signal in neurons.

Previously we demonstrated that PINK1 deficiency is associated with increased basal production of ROS in the mitochondria and cytosol (mediated by NADPH oxidase). We therefore inhibited basal ROS generated by NADPH oxidase using diphenylene iodonium (DPI, 0.5 µM) for 20 minutes prior to these experiments in order to investigate the specific effects of dopamine. Application of 50 µM dopamine to wt and PINK1 ko neurons in the presence of 0.5 µM DPI produced a profound activation of ROS production in both wt and PINK1 ko neurons (100% is the basal rate in untreated wt cells; 196.5±11.5% is the rate of production in dopamine treated cells, n = 54; p<0.001; and 207.5±16.4% is the rate of production in dopamine treated PINK1 ko cells, n = 48; p<0.001; Figure 3a,b,c). The metabolism of dopamine in cells by monoamine oxidase (MAO) results in production of H_2_O_2_, and subsequent generation of free radicals [11]. MAO inhibition by selegiline (20 µM) prevented the dopamine induced ROS production in both wt and PINK1 ko neurons. However in PINK1 ko neurons, the effect of selegiline was smaller than in wt cells (the rate of HEt fluorescence was reduced to 133.2±8.7% in PINK1 ko midbrain neurons (n = 37, Figure 3b) compared to 111.5±7.6% for wt, n = 44, Figure 3a), suggesting that there is a component of the excessive ROS production which is independent of both NADPH oxidase and MAO. As oxidation of hydroethidium is not selective for H_2_O_2_, our method may also capture secondary free radicals (OH^−^, O_2_^−^).

The interaction of dopamine and a PINK1 deficient state results in three sources of free radicals: (1) respiratory chain in mitochondria (2) NADPH oxidase and (3) MAO. We tested the effects of inhibiting each source of free radicals on dopamine induced mitochondrial depolarisation in PINK1 ko neurons. Pre-incubation of cells with the mitochondrially localised antioxidant MitoQ (10 µM) prevented the dopamine induced mitochondrial depolarisation in PINK1 ko neurons (dopamine-induced depolarisation reduced from 58.8±14% to 21.3±3.7%; n = 42; p<0.001; Figure 3e). Application of 0.5 µM DPI also prevented the collapse of Δψ_m_ induced by dopamine (the change in Rh123 fluorescence decreased from 58.8±14% to 20.4±1.9; n = 47; P<0.001; Figure 3d). Furthermore, application of 20 µM selegiline produced an even more protective effect on the Δψ_m_ (dopamine-induced depolarisation reduced from 58.8±14% to 3.2±0.2; n = 68; p<0.0001; Figure 3f). Importantly in the presence of each individual antioxidant there was still a small change in the Δψ_m_ induced by dopamine. In summary all three sources of free radicals contribute to reducing the threshold for dopamine induced PTP opening.

We next investigated the effect of antioxidants and verapamil on the dopamine-induced calcium signal in PINK1 ko astrocytes and neurons (Figure 3g). The effect of dopamine on the calcium signal (measured by the fura-2 ratio) was normalised to 1.0. Selegiline has a selective effect in reducing the dopamine induced calcium signal in astrocytes, while verapamil has a selective effect in reducing the dopamine induced calcium signal in neurons. Note that other antioxidants and inhibitors of ROS production have no effect in neurons or astrocytes. We also confirmed our earlier finding that the dopamine receptor antagonists did not alter the dopamine induced calcium signal in PINK1 ko astrocytes or neurons (Fig. 3g). Previously we demonstrated that the dopamine induced calcium signal in neurons was dependent on dopamine uptake by the dopamine transporter DAT. Here we confirm that the dopamine induced calcium signal in PINK1 deficient neurons is also dependent on uptake of dopamine by DAT, and can specifically be blocked by the inhibitor GYKI 52895 (20 µM) in neurons but not in astrocytes (Fig. 3g).

### Respiratory chain substrates affect dopamine induced PTP opening

**Figure 4:**
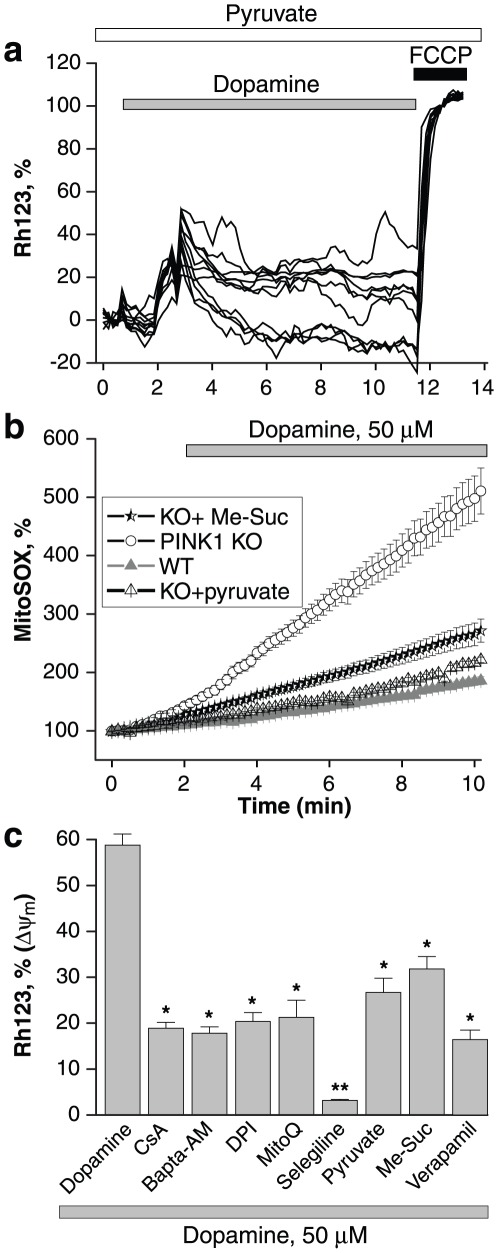
The effect of respiratory chain substrates on mitochondrial membrane potential and ROS generaton. a: Application of 5 mM pyruvate reduced the dopamine induced mitochondrial depolarisation, but did not completely prevent it. b: Dopamine induced a rise in mitochondrial superoxide in PINK1 ko neurons, but not in wt neurons. 5 mM pyruvate and 5 mM Me-succinate both reduced the dopamine-induced mitochondrial ROS production. c: Histogram comparing the reduction of dopamine induced mitochondrial depolarisation by different compounds: the PTP inhibitor CsA, calcium chelator BAPTA-AM, NADPH oxidase inhibitor DPI, mitochondrial antioxidant MitoQ, MAO inhibitor selegiline, respiratory chain substrates pyruvate and me-succinate, and the calcium channel blocker verapamil all resulted in a statistically significant reduction in dopamine induced mitochondrial depolarisation in PINK1 ko neurons.

We have shown that provision of the respiratory chain substrates pyruvate, malate and methyl-succinate to PINK1 deficient cells increases respiratory chain activity, increases the resting mitochondrial membrane potential, and normalises the mechanism used to maintain Δψ_m_ [6]. We sought to determine whether provision of substrates would affect the dopamine induced PTP opening in PINK1 ko neurons. Neurons were incubated with 5 mM pyruvate prior to the application of dopamine. Under these conditions, dopamine did result in a transient but reversible mitochondrial depolarisation (as seen by a rise in the Rh123 fluorescence, Fig. 4a). Cells were then pre-loaded with either 5 mM methyl succinate or 5 mM pyruvate for 40 minutes prior to application of dopamine, and the ROS production measured. Both substrates significantly reduced both the basal rate of mitochondrial ROS production in PINK1 neurons (basal rate in dopamine treated wt cells is 100%; rate in dopamine treated PINK1 ko cells was 376±26%; rate in dopamine treated PINK1 ko cells pretreated with pyruvate was 128.8±9.6%, n = 45; p<0.001; rate in dopamine treated PINK1 ko cells pretreated with me-succinate was 156.4±7.8%; n = 58; p<0.05; Figure 4b). Although pyruvate has a recognised antioxidant effect, methyl succinate does not, and therefore [12] the observed reduction in ROS production is a specific effect of provision of mitochondrial substrates for the respiratory chain.

We tested the ability of different compounds to protect against dopamine induced PTP opening by measuring the degree of dopamine induced depolarisation in dopamine treated PINK1 ko neurons (column 1, Figure 4c), and dopamine treated PINK1 ko neurons preincubated with the following compounds: the PTP inhibitor CsA, calcium chelator BAPTA-AM, NADPH oxidase inhibitor DPI, mitochondrial antioxidant MitoQ, MAO inhibitor selegiline, respiratory chain substrates pyruvate and me-succinate, and the calcium channel blocker verapamil (Figure 4c). PTP inhibition, calcium signal inhibition, and compounds reducing ROS all had a statistically significant effect in reducing dopamine induced mitochondrial depolarisation, but were protective to differing degrees. Pre-incubation of cells with 5 mM pyruvate or 5 mM me-succinate reduced, but did not fully block the effect of dopamine on Δψ_m_ (dopamine-induced depolarisation reduced from 58.8±14% to 26.7±3.1%; n = 61 for pyruvate and to 31.8±2.7%; n = 39 for me-succinate; Figure 4c). We note that the improvement in respiration and reduction in ROS production brought about by mitochondrial substrates did not prevent the mitochondrial depolarisation induced by dopamine, but rather rendered it fully reversible (in contrast to the irreversible PTP opening seen previously in dopamine treated PINK1 ko neurons). Interestingly application of verapamil, which successfully inhibits the dopamine induced calcium signal in neurons [7], was able to partially prevent the dopamine-induced mitochondrial depolarisation in PINK1 ko neurons (Rh123 signal reduced from 58.8±2.4% to 16.7±2.1%, n = 42; p<0.001; Figure 4 C).

### Dopamine induced mitochondrial depolarisation results in cell death

**Figure 5:**
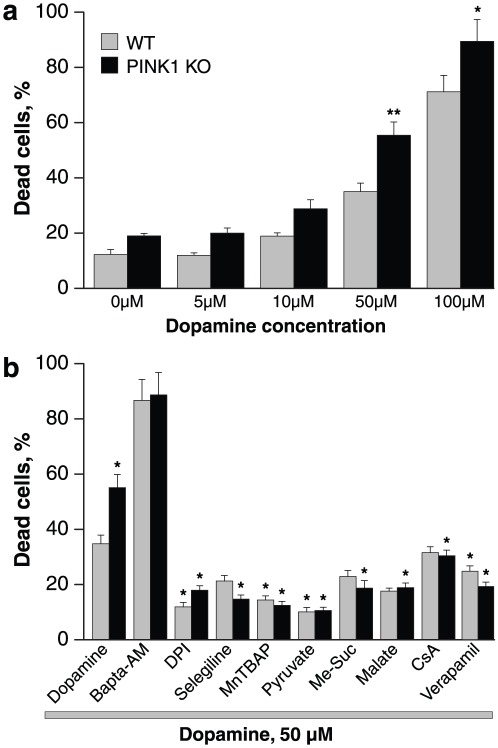
Dopamine induced cell death in PINK1 ko midbrain cultures. a: 24 hr incubation of cells with 50 µM dopamine resulted in a significant increase in cell death in PINK1 ko cultures compared to wt cultures (measured by proportion of cells stained with propidium iodide.). b: Co-application of dopamine with mitochondrial substrates malate, pyruvate, or methylsuccinate, or antioxidants DPI, MnTBAP, or selegiline, or verapamil resulted in a reduction in dopamine induced cell death in both PINK1 ko and wildtype cells. For the wt cultures, the reduction in dopamine induced cell death was statistically significant for DPI, MnTBAP, pyruvate and verapamil (compared to wt cultures treated only with dopamine). For the PINK1 ko cultures, the reduction in dopamine induced cell death was statistically significant for DPI, selegiline, MnTBAP, pyruvate, me-succinate, malate, CsA and verapamil (compared to PINK1 ko cultures treated with dopamine alone).

We first tested the effect of different concentrations of dopamine on cell viability in wt and PINK1 ko midbrain co-cultures of astrocytes and neurons (Figure 5a). Dopamine consistently results in higher levels of toxicity in PINK1 ko cells compared to wt cells across a range of concentrations (5 µM–100 µM). However application of dopamine 50 µM was the first concentration that produced a low level but detectable rise in cell death in wt cells, and significantly different effect in PINK1 ko cells. Therefore this concentration was used throughout the study in order to dissect the mechanism of dopamine induced toxicity in PINK1 deficient cells.

Incubation with dopamine 50 µM for 24 hrs resulted in an increase in cell death in both wt and PINK1 ko cells, although the level of cell death was greater in PINK1 ko cells (34.8±3.1%, n = 13; p<0.001 in wt compared to 55.1±4.8%, n = 16, p<0.001 in PINK1 ko). We next investigated whether the compounds that had been shown to reduce dopamine induced mitochondrial depolarisation had any effect on dopamine induced cell death. Of note, pre-incubation of cells with the inhibitor of PTP, 1 µM CsA, significantly reduced the number of dead cells in PINK1 co-cultures (back from 55.1±4.8% to 30.4±2.1%; p<0.05; Figure 5) confirming that the difference between dopamine induced cell death in wt and PINK1 ko cells is purely due to PTP opening. CsA had no effect in wt cells, confirming that PTP opening is a mechanism unique to PINK1 ko cells.

Preincubation of cells with the intracellular calcium chelator 20 µM Bapta-AM did not protect cells against toxic doses of dopamine: in fact, the % dead cells was higher in both wt and PINK1 ko (86.7±7.6% of dead cells for wt, n = 5 and 88.7±8.1% for ko, n = 5, Figure 5). This is likely to be attributed to the inherent toxicity of Bapta-AM. Pre-incubation of cells with 0.5 µM DPI significantly protected both wt cells (% dead cells reduced from 34.8±3.1 to 11.9±1.6%; n = 6 experiments; p<0.001) and PINK1 ko cells (from 55.1±4.8% to 17.94±1.6%; n = 6; p<0.001; Figure 5). Inhibition of MAO with 20 µM selegiline very effectively protected PINK1 ko cells from dopamine induced cell death (more than 3-fold decrease in % cell death – from 55.1±4.8% to 14.7±1.5%; n = 10; p<0.001; Figure 5). General scavenging of ROS in dopamine-treated cells by MnTBAP significantly reduced cell death in both wt cells (from 34.8±3.1% to 14.4±1.5%; n = 6 experiments; p<0.05) and PINK1 ko cells (from 55.1±4.8% to 12.5±1.4%, n = 6; p<0.001, Figure 5). The mitochondrial substrates pyruvate and methyl succinate, exhibited highly significant protective effects on dopamine induced cell death in both wt and PINK1 ko cells. Thus, 5 mM pyruvate decreased cell death by 5.2-fold in PINK1 ko cells (from 55.1±4.8% to 10.6±1.6%; n = 5; p<0.001; Figure 5) and by 3-fold in wt cells (from 34.8±3.1% to 10.1±1.2%; n = 5; p<0.001). Me-Succinate also protected PINK1 ko cells effectively (% dead cells decreased from 55.1±4.8% to 18.7±2.7%; n = 10 experiments; p<0.05) and much less for wt cells (from 34.8±3.1 to 22.9±2.2%; n = 10; Figure 5). A 2-fold reduction in cell death in PINK1 ko cultures was seen after application of another substrate for mitochondrial compex I – 5 mM malate, confirming that the protective effect of pyruvate and Me-succinate was induced by their effect on the mitochondrial respiratory chain (n = 5 experiments; p<0.05; Figure 5).

Finally inhibition of the dopamine-induced calcium signal in neurons by verapamil led to a significant reduction in the percentage of dead cells in both wt and PINK1 ko cultures (to 24.7±1.9% for wt, p<0.05; and to 19.2±1.6 in PINK1 ko, n = 4; p<0.001; Figure 5).

## Discussion

One of the major questions in PD pathogenesis remains why the SNc dopaminergic neurons are particularly vulnerable to cell death, especially in comparison to the neighbouring VTA neurons, which are also dopaminergic. Several hypotheses for the selective neuronal vulnerability have been proposed [13]: (1) Dopamine itself may be toxic through oxidative stress caused by auto-oxidation or by its metabolism by MAO. During auto-oxidation dopamine is converted to semiquinone and superoxide anion, which reacts with another dopamine molecule to generate semquionone and hydrogen peroxide. During metabolism dopamine is converted to 3,4 dihydroxyphenylacetaldehyde (DOPAA) and to 3,4-dihydroxyphenylacetic acid (DOPAC), with generation of hydrogen peroxide. (2) Dopamine neurons are known to rely on Lv type calcium channels for autonomous pacing, and are therefore exposed to high levels of calcium for prolonged periods of time. Blockade of these calcium channels with concomitant reduction in cytosolic calcium in the SNc dopamine neurons diminishes their sensitivity to PD-associated toxins [14]. Furthermore it has been demonstrated using an electrochemical approach that the cytosolic dopamine concentration is higher in levodopa-treated SNc neurons than levodopa-treated VTA neurons, and that high levels of cytosolic dopamine are sufficient to induce neurotoxicity. Thus the vulnerability of the SNc dopaminergic neurons may be based on a synergistic effect of high levels of dopamine, and higher concentrations of calcium [15].

In this study we have demonstrated that neurons with loss of PINK1 function display enhanced sensitivity to the toxic effects of dopamine. This dopamine-induced toxicity is mediated specifically by permeability transition pore opening in PINK1 deficient cells, which does not occur in wildtype cells. PINK1 deficient neurons exhibit basal mitochondrial dysfunction with impairment of the respiratory chain, increased cytosolic and mitochondrial ROS production, and mitochondrial calcium overload. These factors result in a basal lowered threshold for PTP opening, thus leading to a tendency to induction of the PTP by dopamine.

Permeability transition in the mitochondria is defined as the opening of a proteinaceous pore located in the inner mitochondrial membrane that enables solutes <1500 Da to enter and exit the matrix. Opening of the PTP results in osmotic swelling of the mitochondrial matrix, dissipation of the mitochondrial membrane potential, cessation of ATP synthesis and the release of apoptogenic factors such as cytochrome c, AIF, and Smac/Diablo, that are able to trigger apoptosis. The major inducers of mPTP opening are (1) high cytosolic levels of calcium, resulting in mitochondrial calcium overload and opening of the PTP, and (2) oxidative stress [16]. Other factors include an alkaline matrix pH, reduction of adenine nucleotide pools, and an increase in inorganic phosphate levels and polyphosphate levels [17], [18].

We have demonstrated that in PINK1 deficient cells, dopamine is able to induce opening of the PTP through two major processes that may act synergistically: calcium signalling and ROS production. Dopamine is able to induce a calcium signal in neurons and astrocytes by receptor dependent as well as receptor independent mechanisms. In astrocytes, dopamine induces a calcium signal by MAO-dependent metabolism producing hydrogen peroxide, which activates lipid peroxidation resulting in activation of phospholipase C, release of IP_3_ and a Ca^2+^ signal [7], [8]. This dopamine induced calcium signal is prevented by selegiline in wildtype astrocytes. In neurons, dopamine induces a calcium signal by dopamine uptake via the dopamine transporter resulting in plasmalemmal depolarisation, opening of voltage dependent calcium channels (VDCC) and calcium influx [7], [8]. Thus verapamil and GYKI52895 are able to prevent the dopamine induced calcium signal in wildtype neurons.

In both wildtype and PINK1 deficient cells, we have confirmed that dopamine induces a receptor independent calcium signal i.e the calcium signal is MAO dependent in astrocytes, and VDCC dependent in neurons. Importantly, in wildtype cells, this dopamine induced cytosolic Ca^2^ signal is not associated with significant changes in mitochondrial Ca^2+^. However in PINK1 deficient cells dopamine induces a mitochondrial Ca^2+^ signal, which may occur with or without concomitant visual changes in the cytosolic Ca^2+^. In neurons and astrocytes mitochondria act as the major buffer for cytosolic Ca^2+^, and exist in close relation either to the potential dependent calcium channels, or to the endoplasmic reticulum. Such microdomains result in alterations in calcium signal being visualised in single compartments within the cell, such as the mitochondria but not necessarily in the cytosol [19], [20]. We have previously shown that PINK1 ko neurons have higher basal levels of mitochondrial Ca^2+^ due to an impairment of mitochondrial calcium efflux [6]. In PINK1 deficient neurons therefore, we observe a dopamine induced increase in mitochondrial Ca^2+^ that is sufficient to induce opening of the PTP and cause mitochondrial membrane depolarisation. This dopamine-induced mitochondrial depolarisation can be prevented by the use of calcium chelation in both neurons and astrocytes. Thus the inherent mitochondrial calcium dysregulation caused by loss of PINK1 function, renders neurons particularly vulnerable to repeated dopamine induced calcium elevations.

PTP opening is also regulated by the redox state of mitochondria. We have demonstrated that dopamine induces a change in ROS balance in control cells and PINK1 deficient cells. The mechanism of ROS production in PINK1 deficient cells is three-fold: (1) activation of NADPH oxidase and (2) respiratory chain deficiency both occur in the basal PINK1 deficient state, and (3) MAO activity in metabolism of dopamine. Preventing ROS production through each mechanism is able to prevent the dopamine-induced PTP opening in PINK1 deficient neurons, proving that dopamine induced ROS production is sufficient to induce PTP opening in vulnerable neurons. An increase in Δψm leads to an increase in the threshold of PTP opening [21]. Of note, normalisation of the resting mitochondrial membrane potential and respiratory chain function by the provision of respiratory chain substrates to the cell is able to prevent irreversible dopamine induced PTP opening in PINK1 deficient cells, and results in transient reversible PTP opening only.

It is well recognized that there is considerable interplay and reciprocal interaction between calcium and ROS balance within the mitochondria in both signaling and disease. Therefore dissecting the relative importance of each in dopamine induced PTP opening is challenging. We have previously established that an increase in calcium activates NADPH oxidase with a resultant increase in ROS in PINK1 deficient cells [6]. However we were still able to demonstrate dopamine induced ROS production in the presence of the NADPH oxidase inhibitor DPI, which is not likely to be dependent on calcium. Vice versa, the presence of antioxidants to reduce ROS altered the dopamine induced calcium signal in astrocytes but not in neurons. In astrocytes, the dopamine induced calcium signal is highly dependent on ROS production via MAO [8]. However in neurons the dopamine induced calcium signal is independent of ROS production. Therefore we conclude that the dopamine induced calcium signal and dopamine induced ROS are independently important in the PTP opening in neurons, but act interdependently in the PTP opening in astrocytes.

Our findings have several implications on therapy in PD. First they raise the controversial question whether exogenous dopamine administration in the form of levodopa therapy may contribute to neuronal toxicity. Dopamine has been known to be toxic to neurons in vitro but evidence of dopamine or L-dopa toxicity in vivo models is lacking, and administration of L-dopa to normal rodents or primates does not result in oxidative damage or nigral cell degeneration. Clinical studies in PD patients have also failed to demonstrate an effect of l-dopa toxicity. However imaging studies accompanying clinical trials have suggested that there is a higher rate of decline in ligand uptake in patients on L-dopa compared to placebo (CALM-PD, REAL-PET, ELL-DOPA studies) which raise the possibility that L-dopa administration accelerates cell death [22]. The in vitro data presented here suggests that dopamine is likely to be non-toxic to normal neurons, but in certain vulnerable conditions associated with mitochondrial dysfunction and calcium dysregulation, dopamine may induce toxicity.

This study has further enabled us to deduce novel intervention strategies for neuroprotection in PD. Reduction of oxidative stress within mitochondria using mitochondrial directed antioxidants has been suggested as a therapeutic strategy and this data lends support that it may reduce cell death. However we have found that optimisation of the mitochondrial respiratory function in PINK1 deficient neurons is also highly efficient at preventing dopamine-induced cell death in vitro. The provision of respiratory substrates may therefore represent an intervention that needs to be validated in vivo models of PD. The role of MAO inhibition in PD has been controversial for many years: experimental data has raised the possibility that MAO inhibition may be neuroprotective although the mechanisms remained unclear. We provide evidence that MAO inhibition may prevent dopamine-induced cell death in vitro by limiting ROS production in astrocytes, and by preventing the astrocytic calcium signal induced by dopamine. Finally we have highlighted the role of manipulating neuronal calcium signaling to protect against dopamine induced PTP opening and cell death. Verapamil acts as a calcium channel blocker that reduces the dopamine induced calcium signal in neurons and protects against dopamine induced depolarisation and cell death in susceptible PINK1 deficient neurons.

In summary, we have utilised a genetic primary mammalian model of PD to demonstrate the mechanism of dopamine induced neuronal degeneration. Dopaminergic neurons exposed to dopamine over long periods of time will be susceptible to repeated rises in ROS and calcium. Cells that have reduced ability to handle oxidative stress or calcium fluxes, are unable to handle persistent low doses of dopamine which, over time, may become sufficient to induce PTP opening. PINK1 deficient dopaminergic neurons lack the neuroprotective mechanisms to maintain their calcium and ROS homeostasis and are prone to dopamine toxicity. Of note, manipulation of calcium signaling and the redox state of such cells may represent successful strategies to prevent cell death in PD.

## Materials and Methods

### Cell culture: primary PINK1 ko mouse neuronal model

The PINK1 deficient mice were generated by Lexicon Genetics Inc. (The Woodlands, Texas, USA) (6). For primary mouse midbrain cultures pups were taken at postnatal D2–4. Embryos/pups were obtained either by crossing homozygote animals and comparing same age control animals, or by crossing two heterozygote animals and comparing genotypes within a litter. Animal husbandry and experimental procedures were performed in full compliance with the United Kingdom Animal (Scientific Procedures) Act of 1986. Heads were collected in chilled dissection medium [HBSS without calcium or magnesium, supplemented with 0.45% (v/v) D-(+) glucose, 1 mM sodium pyruvate and 10 mM HEPES pH 7.4]. The whole brain was removed from the skull case in a Petri dish containing chilled dissection medium under sterile conditions, and the meninges were removed. The cortices or midbrain was carefully dissected and transferred to a sterile micro tube containing ∼0.5 ml chilled dissection medium and allowed to settle under gravity. Dissection medium was replaced with 500 µl pre-warmed trypsin solution for 15 minutes at 37°C with gentle agitation halfway through incubation. The trypsination solution was aspirated and the cortices/midbrain washed three times with 500 µl pre-warmed attachment medium [1× Modified Eagles Medium (MEM) with Earles and glutamine (Invitrogen), 1 mM pyruvic acid, 0.45% (w/v) D-(+) glucose (Sigma) and 10% (v/v) heat inactivated fetal bovine serum (Invitrogen)]. The tissue was triturated using sterile fire-polished glass pipettes of differing pore diameter until a smooth suspension was produced. Cells were placed in a humidified CO_2_ incubator (5% CO_2_ in air) at 37°C for 3–4 hrs, before replacing the attachment medium with prewarmed maintenance medium [Neurobasal medium (Invitrogen), 2% (v/v) B27 supplement, 2 mM glutamine, 100 I.U./ml penicillin and 100 I.U./ml streptomycin (Sigma) and 0.45% (v/v) D-(+) glucose]. Once the cells were in maintenance medium, half of the medium was replaced weekly. All live cell imaging experiments were performed between d10-d14 in culture.

Neurones were easily distinguishable from glia: they appeared phase bright, had smooth rounded somata and distinct processes, and lay just above the focal plane of the glial layer.

For measurements of [Ca^2+^]_c_ and [Ca^2+^]_m_, cells were loaded for 30 min at room temperature with either 5 µM fura-2 AM or fluo-4 AM in combination with either 5 µM Rhod-5n, and 0.005% pluronic acid in a HEPES-buffered salt solution (HBSS) composed of (mM): 156 NaCl, 3 KCl, 2MgSO_4_, 1.25 KH_2_PO_4_, 2 CaCl_2_, 10 glucose and 10 HEPES; pH adjusted to 7.35 with NaOH. Previous work using direct and indirect approaches confirmed that the mitochondrial calcium concentration is high in PINK1 ko cells and we have found that the low affinity calcium indicator Rhod-5N is more sensitive to detecting mitochondrial calcium changes in these cells than using a high affinity indicator such as XRhod-1 or Rhod-2. Furthermore at the time of PTP opening the high mitochondrial calcium content will result in the fluorescence of all indicators being fully saturated prior to redistribution on membrane disruption (eg PTP opening). We have therefore used Rhod-5N to detect mitochondrial calcium in this study.

For simultaneous measurement of [Ca^2+^]_i_ and *Δψ_m_*, cells were loaded with fura-2 for 30 minutes, and Rh123 (1 µg/ml; Molecular Probes) was added to the culture during the last 15 min. Cells were washed five times prior to the experiment. Under these loading conditions, Rh123 is non toxic and gives a reliable and reproducible measure of Δψ_m_ through the ‘dequench’ of mitochondrial fluorescence.

For measurement of mitochondrial ROS production, cells were pre-incubated with MitoSOX (5 µM, Molecular Probes, Eugene, OR) for 10 mins at room temperature. For measurement of cytosolic ROS production, dihydroethidium (2 µM) was present in the solution during the experiment to avoid any artefacts related to redistribution of the dye. No preincubation (‘loading’) was used for dihydroethidium to limit the intracellular accumulation of oxidized products.

### Toxicity Experiments

For toxicity assays we loaded cells simultaneously with 20 µM propidium iodide (PI), which is excluded from viable cells but exhibits a red fluorescence following a loss of membrane integrity, and 4.5 µM Hoechst 33342 (Molecular Probes, Eugene, OR), which gives a blue staining to chromatin, to count the total number of cells. A total number of 600–800 cells were counted in 20–25 fields of each coverslip. Each experiment was repeated five or more times using separate cultures.

### Fluorescence Measurements

Fluorescence measurements were obtained on an epifluorescence inverted microscope equipped with a ×20 fluorite objective. [Ca^2+^]_i_ and Δψ_m_ were monitored in single cells using excitation light provided by a Xenon arc lamp, the beam passing through monochromator centred sequentially at 340, 380 and 490 nm (Cairn Research, Kent, UK). Emitted fluorescence light was reflected through a 515 nm long-pass filter to a cooled CCD camera (Retiga, QImaging, Canada). All imaging data was collected and analysed using software from Andor (Belfast, UK). The fura −2 data has not been calibrated in terms of [Ca^2+^]_i_ because of the uncertainty arising from the use of different calibration techniques. Accumulation of Rh123 in polarised mitochondria quenches the fluorescent signal; in response to depolarisation the fluorescence signal is dequenched; an increase in Rh123 signal therefore signals mitochondrial depolarisation. Wherever possible, we have normalised the signals between resting level (set to 0) and a maximal signal generated in response to the uncoupler FCCP (1 µM; set to 100%).

Confocal images were obtained using a Zeiss 510 uv-vis CLSM equipped with a META detection system and a 40× oil immersion objective. The 488 nm Argon laser line was used to excite fluo-4 fluorescence which was measured using a bandpass filter from 505–550 nm. Illumination intensity was kept to a minimum (at 0.1–0.2% of laser output) to avoid phototoxicity and the pinhole set to give an optical slice of ∼2 µm. For MitoSOX or Rhod-5N measurements the 543 nm laser line and 560 nm longpass filter were used. For Het, we generated ratios of the oxidised form (ethidium) excited at 543 nm and measured using a 560 nm longpass filter and the reduced form with excitation at 351 nm, measured between 375–470 nm. All data presented were obtained from at least 5 coverslips and 2–3 different cell preparations.

### Statistical analysis

Statistical analysis and exponential curve fitting were performed using Origin 8 (Microcal Software Inc., Northampton, MA) software. Results are expressed as means ± standard error of the mean (S.E.M.). Paired T-tests were performed for statistical analysis.
